# Nucleocytoplasmic Distribution and Dynamics of the Autophagosome Marker EGFP-LC3

**DOI:** 10.1371/journal.pone.0009806

**Published:** 2010-03-23

**Authors:** Kimberly R. Drake, Minchul Kang, Anne K. Kenworthy

**Affiliations:** 1 Department of Molecular Physiology and Biophysics, Vanderbilt School of Medicine, Nashville, Tennessee, United States of America; 2 Department of Cell and Developmental Biology, Vanderbilt School of Medicine, Nashville, Tennessee, United States of America; Institut Pasteur Korea, Republic of Korea

## Abstract

The process of autophagy involves the formation of autophagosomes, double-membrane structures that encapsulate cytosol. Microtubule-associated protein light chain 3 (LC3) was the first protein shown to specifically label autophagosomal membranes in mammalian cells, and subsequently EGFP-LC3 has become one of the most widely utilized reporters of autophagy. Although LC3 is currently thought to function primarily in the cytosol, the site of autophagosome formation, EGFP-LC3 often appears to be enriched in the nucleoplasm relative to the cytoplasm in published fluorescence images. However, the nuclear pool of EGFP-LC3 has not been specifically studied in previous reports, and mechanisms by which LC3 shuttles between the cytoplasm and nucleoplasm are currently unknown. In this study, we therefore investigated the regulation of the nucleo-cytoplasmic distribution of EGFP-LC3 in living cells. By quantitative fluorescence microscopy analysis, we demonstrate that soluble EGFP-LC3 is indeed enriched in the nucleus relative to the cytoplasm in two commonly studied cell lines, COS-7 and HeLa. Although LC3 contains a putative nuclear export signal (NES), inhibition of active nuclear export or mutation of the NES had no effect on the nucleo-cytoplasmic distribution of EGFP-LC3. Furthermore, FRAP analysis indicates that EGFP-LC3 undergoes limited passive nucleo-cytoplasmic transport under steady state conditions, and that the diffusional mobility of EGFP-LC3 was substantially slower in the nucleus and cytoplasm than predicted for a freely diffusing monomer. Induction of autophagy led to a visible decrease in levels of soluble EGFP-LC3 relative to autophagosome-bound protein, but had only modest effects on the nucleo-cytoplasmic ratio or diffusional mobility of the remaining soluble pools of EGFP-LC3. We conclude that the enrichment of soluble EGFP-LC3 in the nucleus is maintained independently of active nuclear export or induction of autophagy. Instead, incorporation of soluble EGFP-LC3 into large macromolecular complexes within both the cytoplasm and nucleus may prevent its rapid equilibrium between the two compartments.

## Introduction

Macroautophagy (hereafter referred to as autophagy) is a process by which cells degrade intracellular components in order to buffer against starvation conditions, eliminate aggregated cytosolic proteins, and turn over organelles [1]. The process of autophagy involves the formation of double-membrane structures that encapsulate cytosol. These so-called autophagosomes go on to fuse with lysosomes, leading to the degradation of their contents [2].

Microtubule-associated protein light chain 3 (LC3) was the first protein shown to specifically label autophagosomal membranes in mammalian cells [3]. EGFP-LC3 has subsequently become widely used to monitor autophagy by visualizing its recruitment to autophagosomes [4], [5], [6], [7], [8]. The yeast homolog of LC3, Atg8p, is known to function in the formation of autophagosomes in yeast, where it plays a role in membrane tethering and hemifusion during autophagosome formation [9], [10]. The association of LC3 and Atg8p with autophagosome membranes requires several post-translational modifications [3], [11]. The proprotein undergoes cleavage of its C-terminus to form a soluble LC3-I, and is ultimately modified by the attachment of phosphatidylethanolamine to form membrane bound LC3-II [12], [13]. Intra-autophagosomal LC3-II is subsequently degraded [14], [15], whereas cytosolically-localized LC3-II can be released from the autophagosome membrane following delipidation [16].

Although LC3 is currently thought to function primarily in the cytosol, the site of autophagosome formation, EGFP-LC3 is found in the nucleoplasm as well [17], [18], [19], [20]. In principle, given the low molecular weight (∼18 kDa) of the processed forms of LC3, the protein could potentially enter the nucleus by passively diffusing through the nuclear pores even when fused to EGFP, a 27 kDa protein [21]. Interestingly, distinct enrichment of EGFP-LC3 in the nucleus is apparent upon inspection of fluorescence images in a number of published studies, suggesting that instead the entry and exit of the protein may be specifically regulated [8], [17], [18], [19], [20], [22]. Moreover, regulation of the nucleo-cytoplasmic distribution of proteins is increasingly recognized as a control point in the autophagy pathway [23], [24]. However, the nuclear pool of EGFP-LC3 has not been specifically studied in previous reports, and mechanisms by which LC3 shuttles between the cytoplasm and nucleoplasm are currently unknown.

To address this issue, in the current study we investigated the regulation of the nucleo-cytoplasmic transport of soluble EGFP-LC3 using quantitative fluorescence microscopy and photobleaching techniques. We show that soluble EGFP-LC3 is enriched in the nucleus relative to the cytoplasm and that the maintenance of its nuclear enrichment is independent of active nuclear export or induction of autophagy. Instead, our data suggest that incorporation of soluble EGFP-LC3 into large macromolecular complexes within both the cytoplasm and nucleus may prevent its rapid equilibrium between these two subcellular compartments.

## Results

### EGFP-LC3 and tfLC3 are enriched in the nucleus under steady state conditions

**Figure 1:**
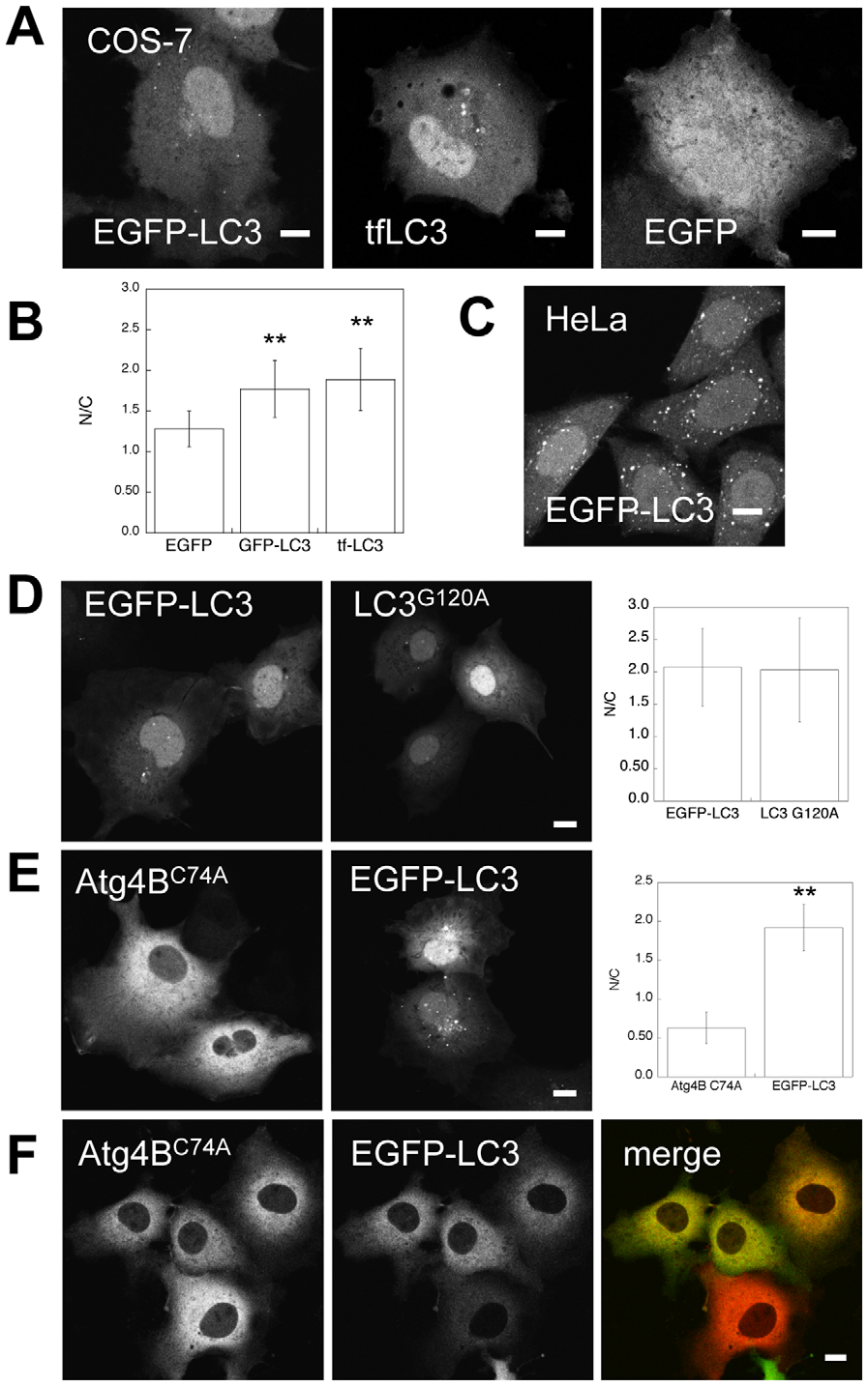
EGFP-LC3 and tfLC3 are selectively enriched in the nucleus under steady state conditions. (**A**) Localization of EGFP, EGFP-LC3, and tfLC3 under steady state conditions in transiently transfected COS-7 cells. (**B**) Quantification of N/C ratios for EGFP, EGFP-LC3, and tfLC3 under steady state conditions was performed as described in the Materials and Methods. Data show the mean ± SD for 40–60 cells from a total of 4–5 independent experiments for each protein. **, p<.0001 compared to EGFP by Student t-test. (**C**) Distribution of EGFP-LC3 in stably transfected HeLa cells. (**D**) (Left) Subcellular distribution of EGFP-LC3 versus EGFP-LC3^G120A^ in COS-7 cells. (Right) Mean N/C ratios were measured for 58-79 cells from 4 independent experiments. (**E**) (Left) Localization of mStrawberry-Atg4B^C74A^ versus EGFP-LC3 when expressed individually in COS-7 cells. (Right) Mean N/C ratios were obtained from 56–61 cells from 3 independent experiments. **, p<.0001 compared to mStrawberry-Atg4B^C74A^ by Student t-test. (**F**) Localization of mStrawberry-Atg4B^C74A^ (red in merged image) and EGFP-LC3 (green in merged image) following their coexpression in COS-7 cells. All scale bars  = 10 µm.

Fluorescence images of cells expressing EGFP-LC3 often show a marked enrichment of the protein in the nucleus compared to the cytoplasm [8], [17], [18], [19], [20]. To examine this nuclear enrichment phenotype in more detail, we transiently expressed two different tagged versions of LC3, EGFP-LC3 and tfLC3 in COS-7 cells, and found that both appeared to be enriched in the nucleus (Figure 1A). To quantify the extent of enrichment of the LC3 fusion proteins in the nucleus, we sampled the average fluorescence intensity of GFP within regions of interest placed within the cytoplasm and nucleus from confocal sections and calculated the nuclear/cytoplasmic ratio for 40–60 cells across 4–5 independent experiments for each protein (Figure 1B). For this analysis, we only considered regions devoid of punctate structures, and thus all references to “cytoplasmic” and “nuclear” EGFP-LC3 refer to the soluble form of the protein. This analysis revealed that both EGFP-LC3 and tfLC3 are enriched in the nucleus ∼1.8 fold. To exclude the possibility that the apparent nuclear enrichment of GFP-LC3 and tfLC3 is an artifact of imaging, we examined the subcellular distribution of EGFP, a soluble 27kDa protein lacking specific targeting information, under identical conditions. Unlike EGFP-LC3, EGFP was equally distributed between the nucleoplasm and cytoplasm (Figure 1A). Quantification verified that the nuclear/cytoplasmic ratio of EGFP-LC3 was in fact significantly higher than that of EGFP (Figure 1B). Similar nuclear enrichment was observed for a mutant of LC3 unable to undergo C-terminal cleavage and lipid conjugation [3], EGFP-LC3^G120A^ (Figure 1D).

We next asked whether enrichment of EGFP-LC3 in the nucleus is specific to COS-7 cells or instead is a more general phenotype. To test this, we examined the localization of EGFP-LC3 in a HeLa cell line stably expressing EGFP-LC3 [18]. Compared to COS-7 cells, a much larger fraction of EGFP-LC3 was constitutively associated with putative autophagosomes in the cytoplasm of the HeLa cell line (Figure 1C). Nevertheless, similar to the COS-7 cells, soluble EGFP-LC3 was clearly present at higher levels in the nucleoplasm than cytoplasm. Thus, the nuclear enrichment of soluble EGFP-LC3 does not appear to be a cell-type specific phenomenon. We therefore next considered potential mechanisms that might regulate the levels of EGFP-LC3 and tfLC3 in the nucleus.

### Coexpression of Atg4B^C74A^ with EGFP-LC3 shifts EGFP-LC3 out of the nucleus

One condition that has been previously noted to change the nucleo-cytoplasmic localization of EGFP-LC3 is co-expression of a mutant form of Atg4B^C74A^, a protease that cleaves the LC3 precursor to produce LC3-I and that catalyzes the delipidation of LC3 [25]. In particular, the presence of a catalytically inactive form of Atg4B, mStrawberry-Atg4B^C74A^, was shown to lead to the sequestration of EGFP-LC3 in the cytoplasm and depletion from the nucleus [25]. When expressed individually in COS-7 cells, mStrawberry-Atg4B^C74A^ was found predominantly in a soluble cytoplasmic pool, whereas EGFP-LC3 was distributed in both the nucleus and cytoplasm (Figure 1E). However, when we cotransfected COS-7 cells with EGFP-LC3 and mStrawberry-Atg4B^C74A^, a marked shift in the localization of EGFP-LC3 out of the nucleus and into the cytoplasm was observed (Figure 1F). These data confirm that the nucleocytoplasmic distribution of EGFP-LC3 is subject to regulation by protein-protein interactions in the cytoplasm [25]. We therefore next tested the role of other mechanisms, such as active nuclear transport, that could potentially modulate EGFP-LC3's nucleocytoplasmic distribution.

### LC3 contains a putative NES, but the nucleo-cytoplasmic distribution of EGFP-LC3 is maintained independently of active nuclear export

**Figure 2:**
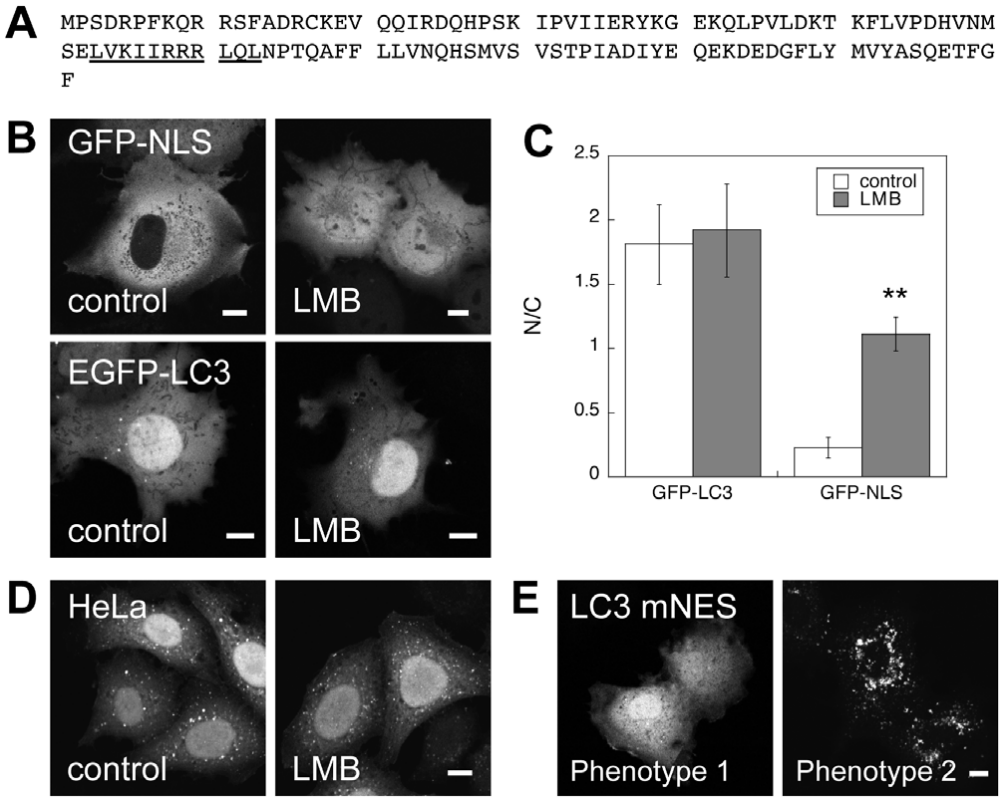
LC3 contains a putative NES, but remains enriched in the nucleus following blockade of nuclear export or mutation of the NES. (**A**) Sequence of human LC3. The predicted leucine-rich nuclear export signal is underlined. (**B**) Effect of LMB, a specific inhibitor of nuclear export, on the distribution of EGFP-LC3 or Rev(68–90)GFP_2_-cNLS. COS-7 cells expressing EGFP-LC3 or Rev(68–90)GFP_2_-cNLS were treated with 20 nM LMB for 3 h at 37°C. The cells were then shifted onto the microscope stage for imaging. Scale bar  = 10 µm. (**C**) Quantification of the mean intensity of EGFP-LC3 and Rev(68–90)GFP_2_-cNLS in the nucleoplasm versus the cytoplasm of COS-7 cells under control conditions and following LMB treatment. Data show the mean ± SD for 20 cells from 2 independent experiments. **, p<.0001 compared to control by Student t-test. (**D**) Distribution of EGFP-LC3 in stably transfected HeLa cells following treatment with 20 nM LMB or vehicle for 3 h at 37°C. Scale bar  = 10 µm. (**E**) Distribution of EGFP-LC3 mNES mutant in COS-7 cells. Representative images showing the two major phenotypes observed are shown. Scale bars  = 10 µm.

Nucleocytoplasmic transport can be controlled in a variety of ways including regulation of the nuclear pore complex, transport receptors, and cargo themselves [21]. One specific mechanism by which EGFP-LC3 could become enriched in the nucleus is through regulated nucleocytoplasmic shuttling. Cargo molecules that are actively transported through the nuclear pore complex contain signals that are recognized by transport receptors. Recognition for import into the nucleus is mediated by a nuclear localization signal (NLS) whereas export is determined by a nuclear export signal (NES) [21]. We analyzed the sequence of human microtubule-associated proteins 1A/1B light chain 3A precursor for NLS and NES sequences. No known NLS sequences were identified. However, a putative NES was identified using an algorithm that predicts leucine-rich nuclear export signals [26]. This predicted NES is located at residues 63 to 73 of human LC3 (Figure 2A). The presence of this NES suggests that the nuclear transport of LC3 may indeed be actively regulated.

As an initial test of whether the transport of EGFP-LC3 out of the nucleus is actively regulated, we examined the effect of inhibition of nuclear export. CRM-1 is a protein required for the nuclear export of proteins containing an NES [27]. Leptomycin B (LMB) specifically inhibits nuclear export by alkylating CRM-1 [28]. As a positive control for the effect of LMB, we examined the distribution of a shuttling reporter protein containing a classic NLS and NES, Rev(68–90)GFP_2_-cNLS. [29]. In COS-7 cells, Rev(68–90)GFP_2_-cNLS had a predominant cytoplasmic distribution under steady state conditions, suggesting that in this cellular environment the NES is dominant over the NLS (Figure 2B). Treatment of COS-7 cells with 20 ng/ml LMB for 3 h [30] caused a significant accumulation of Rev(68–90)GFP_2_-cNLS in the nucleus. In contrast, no change in the nuclear/cytoplasmic ratio of EGFP-LC3 was observed in the presence of LMB (Figure 2B, C). LMB treatment similarly had no effect on the subcellular distribution of EGFP-LC3 in HeLa cells (Figure 2D). This suggests that maintenance of the steady state localization of EGFP-LC3 does not require active export from the nucleus.

Beclin-1 is known to contain a leucine-rich NES that is necessary for its function in autophagy [23]. Mutation of the NES shifts beclin-1 from a primarily cytoplasmic to nuclear localization [23]. We used a similar strategy to further test for a role of active nuclear transport in control of the steady state levels of EGFP-LC3 in the nucleus versus cytoplasm, by generating a mutant form of EGFP-LC3 (EGFP-LC3 mNES) in which three leucines in the putative NES were mutated to alanine (L63A L71A L73A). Two phenotypes were observed in cells expressing EGFP-LC3 mNES (Figure 2E). In the majority of cells, the protein was diffusely distributed in the cytoplasm and nucleoplasm (Figure 2E, left panel). In a smaller fraction of cells, the NES mutant protein was associated with punctate structures in the cytoplasm, which presumably correspond to autophagosomes and/or protein aggregates (Figure 2E, right panel). Thus, the putative NES of LC3 does not functionally direct the protein out of the nucleus in a CRM-1 dependent fashion under steady state conditions.

### The distribution of EGFP-LC3 is modestly affected by microtubule disruption

**Figure 3:**
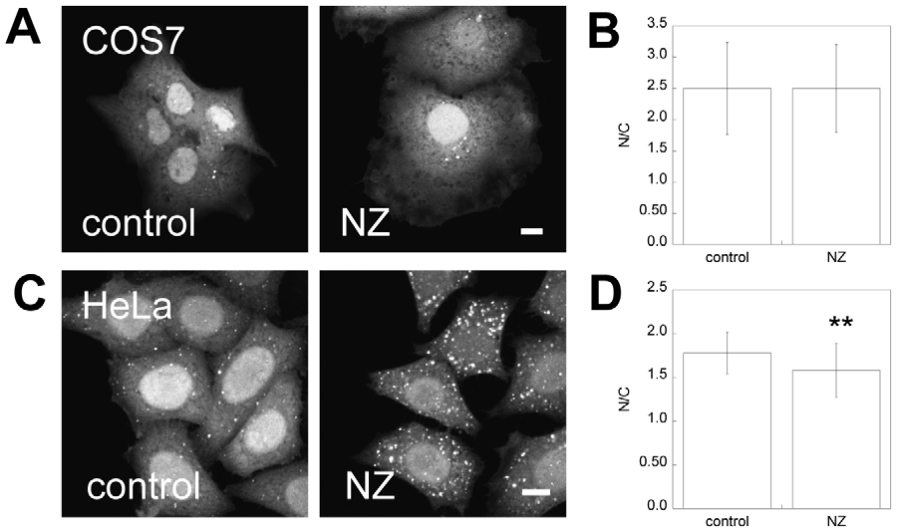
The nucleocytoplasmic distribution of EGFP-LC3 is modestly affected by microtubule disruption. Cells were subjected to microtubule disruption with 5 µg/ml NZ or mock-treated (“control”) and fixed prior to imaging as described in the Materials and Methods. (**A**) Effect of NZ on the distribution of EGFP-LC3 in COS-7 cells. (**B**) Quantification of N/C for soluble EGFP-LC3 in mock-treated or NZ-treated COS-7 cells. Data show the mean ± SD for 28–30 cells from 2 independent experiments. (**C**) Effect of NZ on the distribution of GFP-LC3 in HeLa cells. (**D**) As in B except data are for EGFP-LC3 in HeLa cells. Data show the mean ± SD for 62–72 cells from 2 independent experiments. Scale bar  = 10 µm.

LC3 is a microtubule binding protein [31], and autophagosome formation and dynamics are also known to be microtubule dependent [20]. We therefore asked whether the nucleo-cytoplasmic distribution of EGFP-LC3 requires intact microtubules. To test this, we utilized nocodazole (NZ) treatment to disrupt microtubules [32]. Under these conditions, in COS-7 cells the distribution of EGFP-LC3 was unaltered compared to cells subjected to mock NZ treatment (Figure 3A,B). In HeLa cells, NZ treatment resulted in a visible increase in the number and fluorescence intensity of EGFP-LC3 puncta in the cytoplasm (Figure 3C). Image analysis showed that the soluble pool of protein had a lower N/C ratio in NZ-treated cells than mock-treated cells (Figure 3D). Therefore, in HeLa cells, microtubule disruption influences the N/C ratio of EGFP-LC3, but do not appear to be required to maintain a pool of EGFP-LC3 within the nucleus.

### EGFP-LC3 is transported into and out of the nucleus at a very slow rate

**Figure 4:**
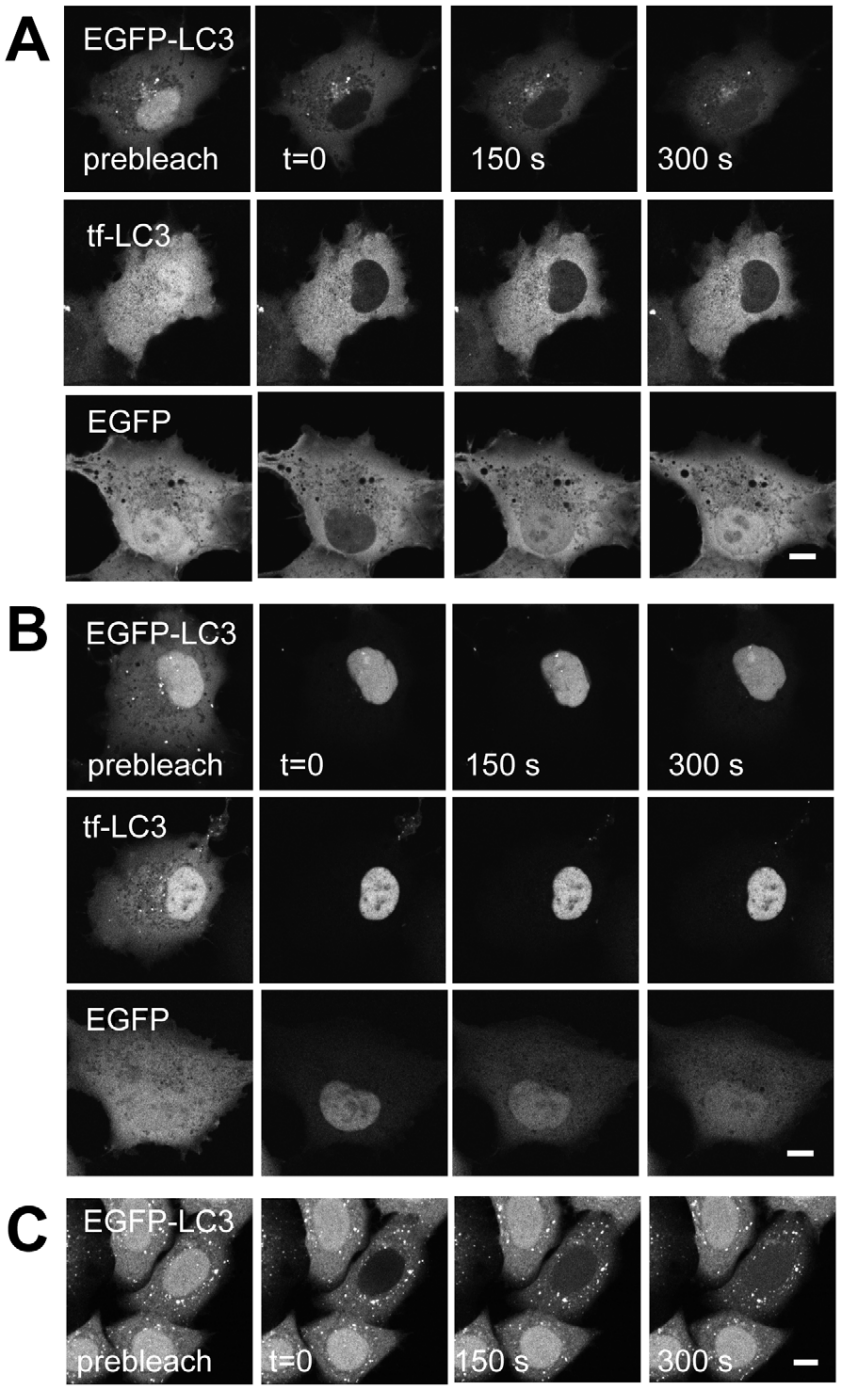
The kinetics of nuclear import and export of EGFP-LC3 and tfLC3 are significantly slower that that of EGFP as assessed by photobleaching. Photobleaching experiments of the entire nucleus or entire cytoplasm were performed at 37°C. (**A**) Images from a representative nuclear photobleaching experiment measuring the kinetics of nuclear import of EGFP-LC3, tfLC3, and EGFP in COS-7 cells. (**B**) As in A, except the cytoplasmic pool of proteins were photobleached. (**C**) Images from a representative nuclear photobleaching experiment of EGFP-LC3 in HeLa cells. Bars, 10 µm.

Although EGFP-LC3 does not appear to undergo actively regulated or induced transport, it is still possible that the protein moves across the nuclear envelope by passive diffusion. To determine if this is the case, we used FRAP to determine the kinetics of EGFP-LC3's nucleocytoplasmic transport [30], [33], [34]. For these experiments, we used a confocal microscope to selectively photobleach the fluorescent protein in the nucleus or cytoplasm by repetitively scanning the region using 100% laser power. A series of images was then collected over time using low laser illumination in order to monitor recovery of the fluorescent protein into the bleached region (Figure 4). For comparison, we measured the transport kinetics of EGFP as an example of a protein that undergoes passive diffusion through nuclear pores [35].

Photobleaching measurements of nuclear import revealed that EGFP equilibrated to its original distribution within 5 min (Figure 4A). This is consistent with the ability of EGFP to diffuse passively through nuclear pores [30], [36]. However, recovery of EGFP-LC3 into the nucleus occurred considerably more slowly than that of EGFP (Figure 4A). The nuclear export rate of EGFP-LC3, measured by photobleaching the cytoplasmic pool of the protein, was also markedly slower than that of EGFP (Figure 4B). Similar results were obtained for tfLC3 (Figure 4A, B). Recovery of EGFP-LC3 in the nucleus was visible within 300 s, but still had not returned to steady state values in the stable EGFP-LC3 expressing HeLa cell line (Figure 4C). Thus, transport of the LC3 fusion proteins into and out of the nucleus occurs more slowly that than of EGFP under steady state conditions, ruling out a model in which rapid passive transport contributes significantly to its steady state distribution.

### The diffusional mobility of EGFP-LC3 in the nucleus and cytoplasm suggests it undergoes transient binding events and/or associates with a high molecular weight complex

We next considered possible mechanisms that could account for the impeded movement of EGFP-LC3 and tfLC3 into and out of the nucleus. The transport of some proteins between the cytoplasm and nucleus is controlled by interactions with binding partners in each compartment rather than active transport across the nuclear pore [34], [36]. The diffusional mobility of proteins can be used to test whether they interact with cellular components such as chromatin or diffuse as part of a high molecular weight complex [37], [38]. To test whether this is the case for EGFP-LC3 and tfLC3, we used fluorescence recovery after photobleaching (FRAP) to measure the diffusional mobility of the protein within the nucleus and cytoplasm [39], [40], [41]. In these experiments, a confocal microscope was used to monitor the fluorescence intensity in a small region of interest (ROI) within the nucleus or cytoplasm before and after irreversibly photobleaching the molecules within the ROI by repetitively scanning at 100% laser power. Following the photobleach, non-fluorescent molecules within the ROI exchange with fluorescent molecules in the surrounding area by diffusion. Note that this experiment differs from the measurement of nuclear import described above in that only a small region is bleached within the nucleus. Thus, fluorescence recovery occurs by lateral diffusion and not transport across the nuclear membrane, which occurs over longer times than the characteristic time of diffusional recovery.

FRAP data can be characterized by two parameters, an apparent diffusion coefficient (*D*) which provides a measure of the mean squared displacement of the diffusing molecule per unit time, and a mobile fraction (Mf) a measure of the fraction of molecules that are free to diffuse over the time course of the experiment [37]. In such experiments, monomeric proteins that do not interact with any cellular components, such as EGFP [42], [43], should have a high mobile fraction and a diffusion coefficient consistent with their size and shape. For such molecules, *D* can be directly calculated according to the Stokes-Einstein equation, *D*  =  (κ_B_ *T*)/(6πη*r*), where κ_B_ is the Boltzmann constant, *T* absolute temperature, η the viscosity, and *r* the Stokes radius of the diffusing molecule. In contrast, proteins that transiently bind cellular structures or that diffuse as part of a large complex will diffuse more slowly than predicted for their size and shape [38], [42]. For example, the diffusion of transcription factors is slowed by transient binding to DNA [42], [44]. Finally, proteins that are tightly bound to immobile structures such as chromatin are predicted to have a slow diffusion coefficient and/or low mobile fraction compared to a freely diffusing soluble protein such as EGFP. Such behavior has been observed for histone 2B [45].

**Figure 5:**
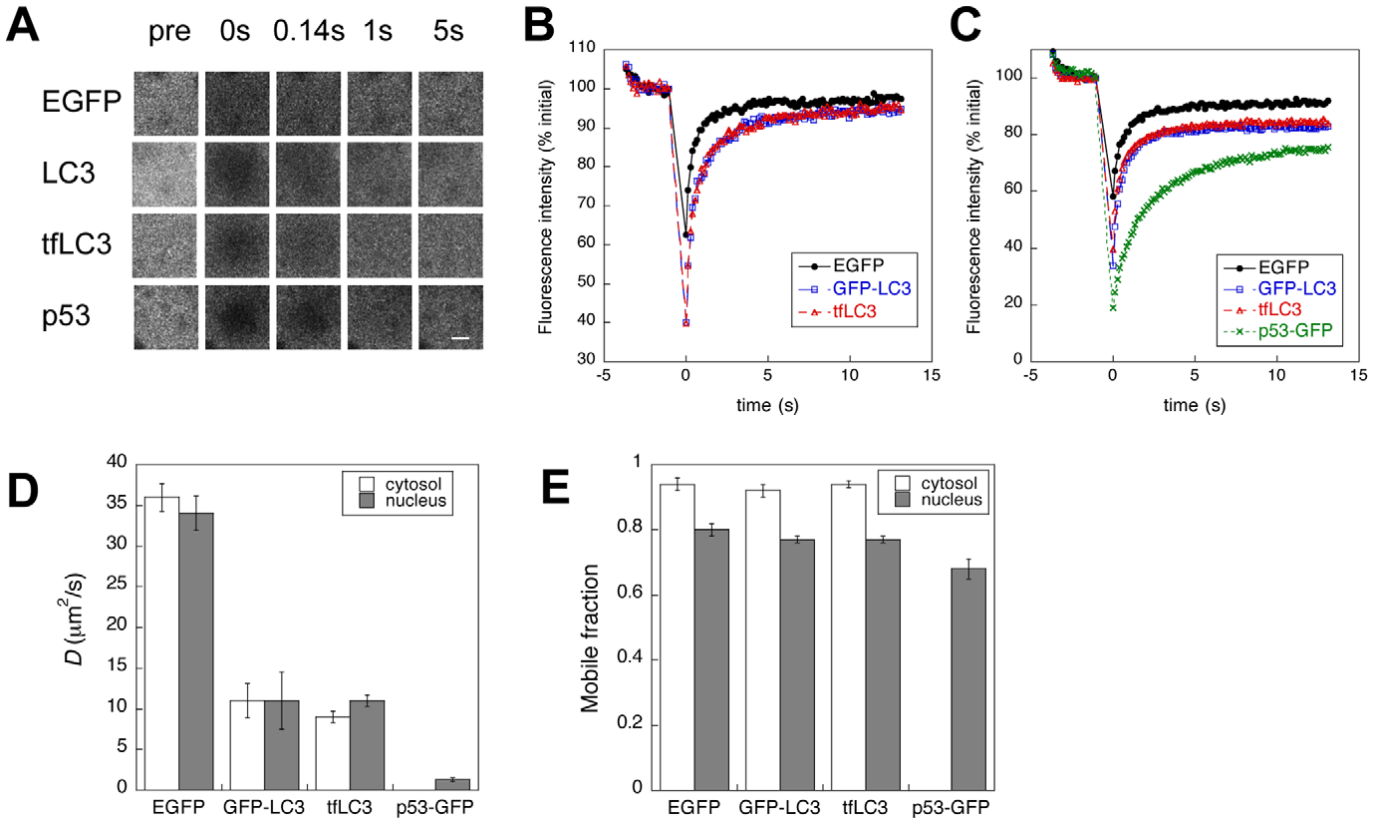
EGFP-LC3 and tfLC3 diffuse more slowly than predicted by their molecular weights. COS-7 cells were transfected with the indicated constructs. The following day, confocal FRAP experiments were performed at 37°C as described in the Materials and Methods. (**A**) Representative images collected during a FRAP experiment in COS-7 cells. Data are shown for measurements in the nucleus. Scale bar  = 2 µm. (**B**) Recovery curves for EGFP (black circles), EGFP-LC3 (blue squares), and tfLC3 (red triangles) in the cytoplasm of COS-7 cells. Data show the mean values for 10 cells from a representative experiment. For clarity, error bars are not shown. (**C**) Recovery curves for EGFP (black circles), EGFP-LC3 (blue squares), and p53-GFP (green crosses) in the nucleus of COS-7 cells. Data show the mean ± SE for 10 cells from a representative experiment. For clarity, error bars are not shown. (**D**) Effective diffusion coefficients for EGFP, EGFP-LC3, tfLC3, and p53-GFP in the cytosol (white bars) and nucleus (gray bars) of COS-7 cells. Data show the mean ± SD for 30 cells from a total of 3 independent experiments. (**E**) Mobile fractions for EGFP, EGFP-LC3, tfLC3, and p53-GFP in the cytosol (white bars) and nucleus (gray bars) of COS-7 cells. Data show the mean ± SD for 30 cells from a total of 3 independent experiments.

To analyze the diffusional mobility of EGFP-LC3 and tfLC3, we bleached a small circular region of interest located either in the nucleoplasm or the cytoplasm, and imaged the bleach ROI and the area immediately surrounding it during the recovery (Figure 5). The resulting recovery curves were then fit with a model that corrects for diffusion during the bleach as described in the Materials and Methods [46]. As controls, we performed parallel experiments for EGFP and p53-GFP, a protein that transiently binds to DNA [42], [44]. Because EGFP-LC3 is only ∼20 kDa larger than EGFP, its *D* should be quite similar to that of EGFP if it behaves as a freely diffusing monomer. In contrast, we found that *D* for both EGFP-LC3 and tfLC3 in the nucleus was substantially slower than that of EGFP, but faster than that of p53-GFP (Figure 5C, D). In the cytoplasm, *D* of EGFP-LC3 and tfLC3 was again significantly (∼3 fold) slower than that of EGFP (Figure 5B, D). Moreover, *D's* for EGFP-LC3 and tfLC3 diffusion were similar to one another in both the nucleus and cytoplasm of COS-7 cells (Figure 5). In addition, Mf of EGFP-LC3 and tfLC3 were nearly as high as for EGFP, suggesting that LC3 is not stably bound to immobile structures within the cell (Figure 5E).

To determine whether the slow diffusion of EGFP-LC3 was an artifact of its overexpression in COS-7 cells, we repeated the FRAP experiments in the stable EGFP-LC3 expressing HeLa cell line. As for the case of COS-7 cells, we observed that *D* for EGFP-LC3 was slower than expected for a monomeric protein of its molecular weight. However, in HeLa cells we also observed a significantly higher value of *D* in the nucleus (4.5±0.3 µm^2^/s, N = 30 cells from 3 independent experiments) versus cytoplasm (3.1±0.1 µm^2^/s, N = 21 cells from 3 independent experiments). The Mf for nuclear and cytoplasmic EGFP-LC3 were identical in the HeLa cells (0.77±0.06).

Taken together, these FRAP data suggest that EGFP-LC3 does not exist as a freely diffusing monomer in either the cytoplasm or nucleoplasm, but rather is either incorporated into a macromolecular complex or reversibly binds cellular components.

### Analysis of the nuclear/cytoplasmic distribution and diffusion of EGFP-LC3 following induction of autophagy

**Figure 6:**
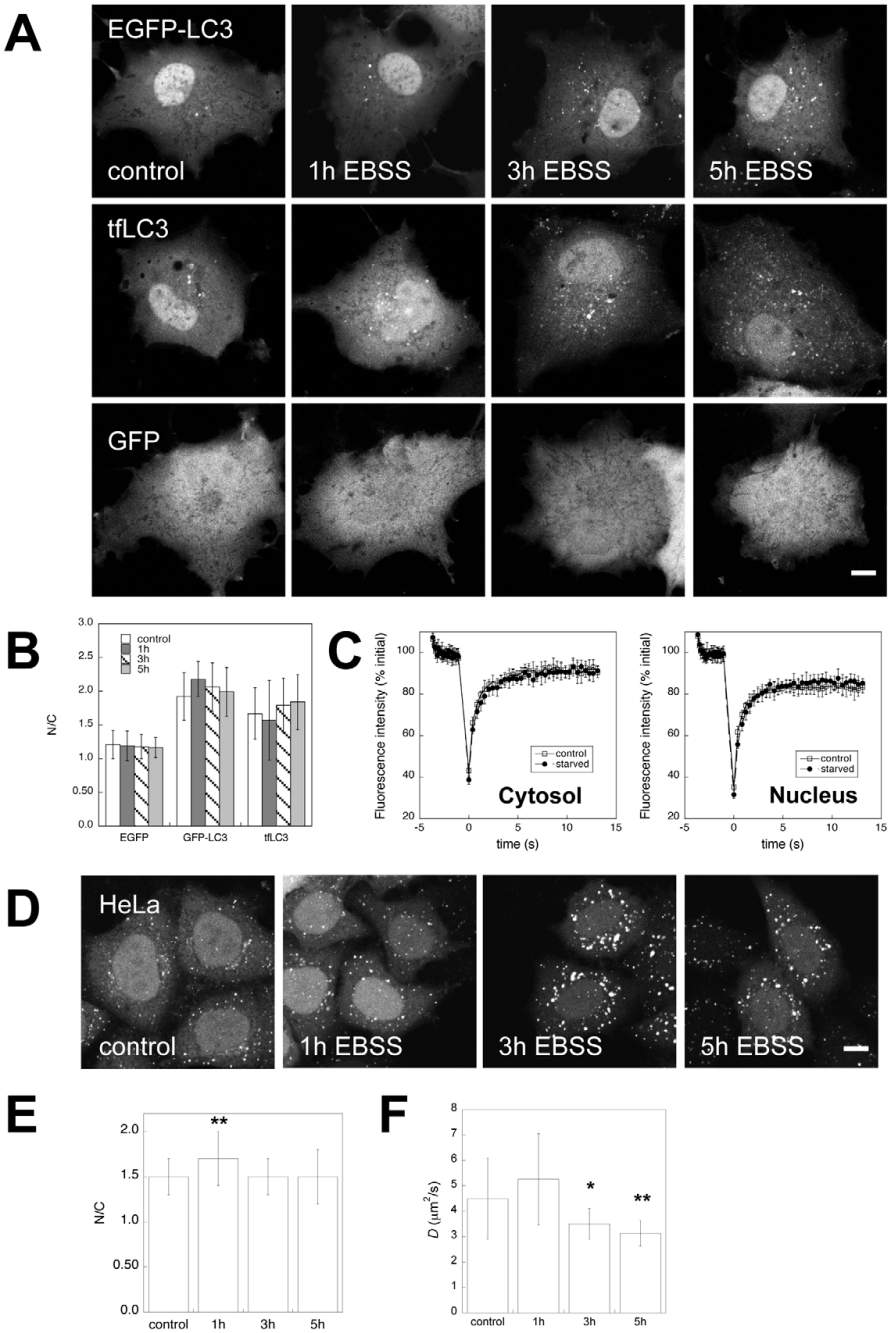
Effect of amino acid starvation on the nuclear/cytoplasmic ratio and diffusional mobility of EGFP-LC3. (**A**) Effect of amino acid starvation on the subcellular localization of EGFP-LC3, tfLC3, and EGFP in live COS-7 cells. Bar, 10 µm. (**B**) Quantification of the mean intensity of EGFP, EGFP-LC3, and tfLC3 in the nucleoplasm versus the cytoplasm under control conditions and following amino acid starvation in COS-7 cells. Data show the mean ± SD for 20-30 cells from 2–3 independent experiments for each protein. (**C**) Representative FRAP curve for EGFP-LC3 in the cytoplasm or nucleus of untreated COS-7 cells (“control”, open squares) and COS-7 cells incubated for 5 h in EBSS (“starved”, closed circles). FRAP experiments were performed as described in the Materials and Methods. Data show the mean± SD for 8–10 cells from a representative experiment. For clarity, error bars are only shown for every third datapoint after the bleach. (**D**) Effect of amino acid starvation on the localization of EGFP-LC3 in live HeLa cells. Bar, 10 µm. (**E**) Quantification of the N/C ratio of EGFP-LC3 under control conditions and following amino acid starvation in live HeLa cells. Data show the mean ± SD for 81–117 cells from 3 independent experiments. (**F**) Effective diffusion coefficients for nuclear EGFP-LC3 in HeLa cells under control conditions or following amino acid starvation. Data show the mean ± SD for 20–30 cells from 3 independent experiments. *, p<.01 compared to control, and **, p<.0006 compared to control by Student t-test.

The finding that EGFP-LC3 and tfLC3 are enriched in the nucleus under steady state conditions additionally raises the question of whether their nuclear-cytoplasmic distribution is physiologically regulated. To test this, we examined the distribution of EGFP-LC3 and tfLC3 during the induction of autophagy by amino acid starvation and rapamycin treatment. As expected [14], with increasing time of starvation the number of soluble EGFP-LC3 and tfLC3-positive putative autophagosomes increased in both COS-7 (Figure 6A) and HeLa cells (Figure 6D). In HeLa cells, the amount of EGFP-LC3 associated with autophagosomes also increased relative to the levels of soluble protein (Figure 6D; note the decreased diffuse fluorescence relative to the punctate staining at 5 h and 5 h relative to control.)

**Figure 7:**
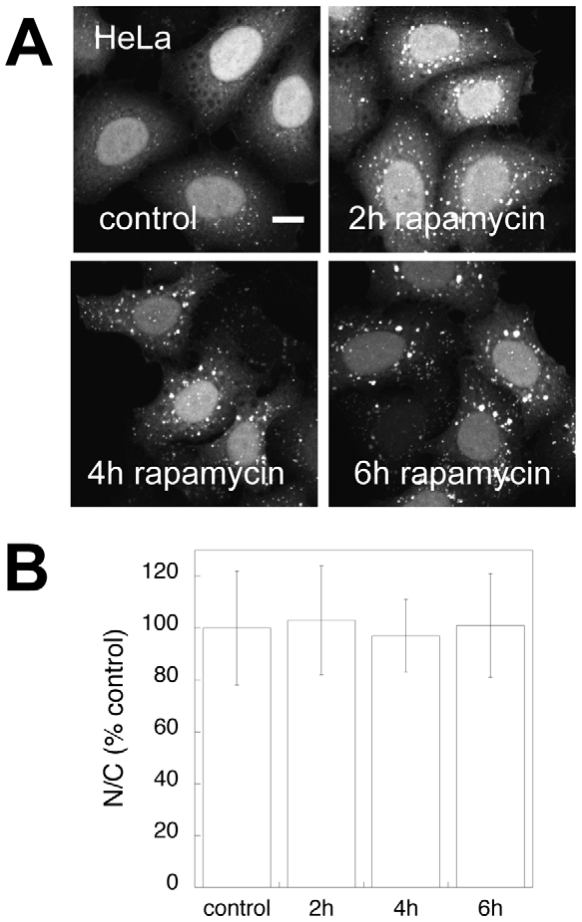
Effect of rapamycin on the nucleo-cytoplasmic distribution of EGFP-LC3. (**A**) Effect of rapamycin treatment or incubation with vehicle (control) on the distribution of EGFP-LC3 in fixed HeLa cells. Bar, 10 µm. (**B**) Quantification of the N/C ratio of EGFP-LC3 following rapamycin treatment versus treatment with vehicle (control) in fixed HeLa cells. To correct for differences in basal N/C values across experiments, data for rapamycin treated cells were normalized to control values for a given experiment. Data represent the mean ± SD for 98–113 cells from 3 independent experiments.

We next quantified the fluorescence levels of soluble EGFP-LC3 from regions of interest placed within in the cytoplasm and nucleoplasm to assess the nucleo/cytoplasmic ratio. In COS-7 cells, the nuclear-cytoplasmic ratio of soluble EGFP-LC3 and tfLC3 remained identical within error to control values, even after 5 h of nutrient starvation (Figure 6B). In contrast, in the stable HeLa cell line, soluble EGFP-LC3 showed a transient increase in nuclear-cytoplasmic ratio following 1 h of amino acid starvation in 2 out of 3 experiments (Figure 6D, E). When autophagy was induced by rapamycin treatment of HeLa cells, the number and brightness of autophagosomes again increased as reported by EGFP-LC3 labeling (Figure 7A). Quantitative analysis of multiple cells indicated that the nucleo-cytoplasmic levels of soluble EGFP-LC3 remained steady compared to controls following rapamycin treatment (Figure 7B). Thus, the induction of autophagy led to a small, transient increase in the levels of soluble EGFP-LC3 in the nucleus versus cytoplasm of HeLa cells at early times following upregulation of autophagy by starvation, but not by rapamycin treatment.

We also wondered if the diffusional mobility of EGFP-LC3 within the nucleus or cytoplasm is altered by upregulation of autophagy. To test this, we performed FRAP experiments of the nuclear and cytoplasmic pools of soluble EGFP-LC3 in COS-7 cells under control conditions and following 5 h of amino acid starvation. The resulting recovery curves were very similar for control and starved cells (Figure 6C). We repeated this analysis in the HeLa cells, but in this case restricted our analysis to nuclear protein as the large number of autophagosomes in the cytoplasm interfered with the FRAP experiments. Interestingly, *D* for EGFP-LC3 in the nucleus of HeLa cells underwent a small but significant decrease as a function of starvation time (Figure 6F). This implies that the same mechanisms that retard the lateral mobility of EGFP-LC3 in the nucleus under steady state conditions are preserved and/or even further upregulated when autophagy is induced by amino acid starvation.

### Endogenous LC3 can also be detected in both the nucleus and cytoplasm under steady state conditions

In the experiments described above, we focused exclusively on the properties of GFP-tagged versions of LC3. There is currently conflicting evidence as to whether endogenous LC3 localizes to the nucleus in addition to the cytoplasm [47], [48]. However, some previous immunofluorescence studies of endogenous LC3 utilized digitonin permeabilization, a condition that preferentially permeabilizes the plasma membrane while leaving the nuclear envelope intact [49]. As a result, the presence of LC3 in the nucleus could have previously gone undetected. We therefore examined the distribution of endogenous LC3 in COS-7 cells by immunofluorescence microscopy.

**Figure 8:**
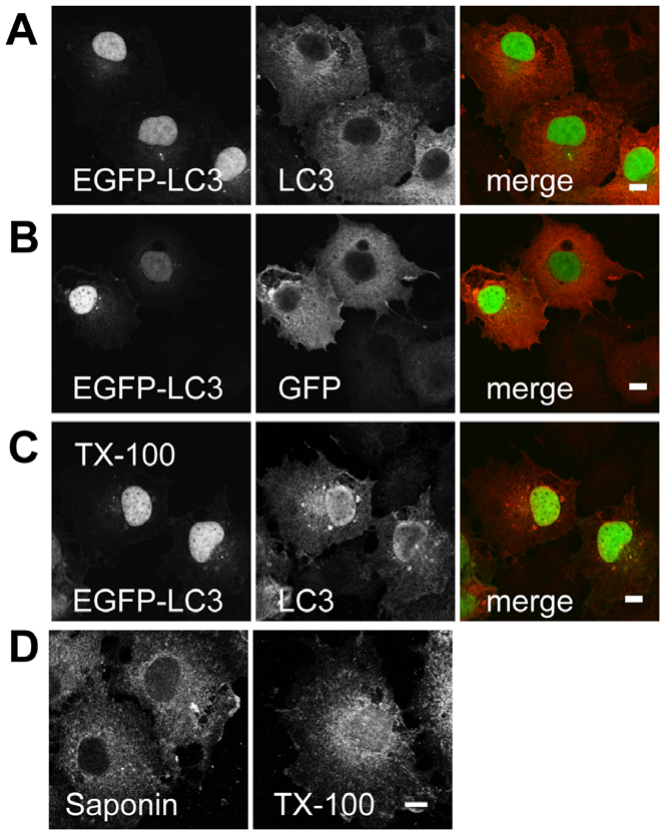
Detection of exogenous and endogenous LC3 in the nucleoplasm by immunostaining. (**A**) COS-7 cells expressing EGFP-LC3 were fixed in 3.7% PFA, permeabilized with 0.1% saponin, and processed for immunostaining for LC3 as described in the Materials and Methods. In the merged images, GFP fluorescence is shown in green and antibody staining in red. (**B**) As in (A), except cells were labeled with an anti-GFP antibody. (**C**) COS-7 cells expressing EGFP-LC3 were fixed in PFA and permeabilized with TX-100, and stained for LC3 as described in the Materials and Methods. In the merged images, GFP fluorescence is shown in green and antibody staining in red. (**D**) Untransfected COS-7 cells were fixed and permeabilized using saponin or TX-100 as described in the Materials and Methods and then immunostained for endogenous LC3. Bars, 10 µm.

To validate conditions that enable detection of LC3 in the nucleus, we first immunostained cells expressing EGFP-LC3 (Figure 8). In saponin permeabilized cells, labeling by the anti-LC3 antibody was confined to the cytoplasm, despite the presence of EGFP-LC3 in the nucleus (Figure 8A). This was not due to masking of the epitope detected by the LC3 antibody in the nucleus, as similar results were obtained using a GFP antibody (Figure 8B). We therefore expanded the fixation and permeabilization conditions to determine under what conditions nuclear EGFP-LC3 could be detected, and found PFA/TX-100 permeabilization allowed for visualization of both cytoplasmic and nuclear EGFP-LC3 (Figure 8C).

Having established conditions to detect both nuclear and cytoplasmic staining of exogenous EGFP-LC3 by immunofluorescence microscopy, we next evaluated the localization of endogenous LC3 in COS-7 cells. In cells subjected to PFA/TX-100 fixation and permeabilization, staining of endogenous LC3 was observed in both the cytoplasm and nucleus (Figure 8D). Thus, like EGFP-LC3, endogenous LC3 also appears to reside within the nucleus under steady state conditions.

## Discussion

### Mechanisms involved in controlling the nucleo-cytoplasmic distribution of EGFP-LC3

Although EGFP-LC3 is a marker of autophagosomes, a substantial fraction of the protein is often present in the nucleus as well [17], [18], [19], [20], [22]. Moreover, levels of soluble EGFP-LC3 in the nucleus often appear higher than levels of soluble EGFP-LC3 in the cytosol, suggesting that the protein does not simply passively equilibrate across the nuclear envelope. In the current study, we therefore investigated how the nucleo-cytoplasmic transport of EGFP-LC3 from the nucleus is regulated.

We first verified that, consistent with published images, soluble EGFP-LC3 indeed is enriched in the nucleoplasm relative to the cytoplasm in two cell types, transiently transfected COS-7 and stably transfected HeLa, as assessed by quantitative fluorescence microscopy. In addition, we confirmed recently published findings that EGFP-LC3 is sequestered in the cytoplasm by coexpression of a mutant form of Atg4B, a protease that cleaves the LC3 precursor to produce LC3-I and that catalyzes the delipidation of LC3 [25]. Indeed, of all the conditions we examined, only coexpression of mStrawberry-Atg4B^C74A^ and EGFP-LC3, resulted in a complete loss of EGFP-LC3 from the nucleus. Thus, protein-protein interactions represent one mechanism that controls the nucleocytoplasmic distribution of EGFP-LC3.

Since LC3 is a microtubule binding protein, we next tested whether intact microtubules are required to maintain the nucleocytoplasmic distribution of soluble EGFP-LC3. We found that microtubule disruption with NZ had little effect on the localization of the protein in COS-7 cells, but modestly decreased the nucleo-cytoplasmic ratio of the protein in HeLa cells, perhaps due to increased association of the protein with autophagosomes under these conditions as discussed in more detail below.

We next tested whether active transport plays a role in regulating LC3's steady state distribution. We identified a predicted leucine-rich NES within residues 63–73, a stretch of residues contained with the third alpha helix of LC3 as determined by both x-ray crystallography and NMR [50], [51]. We predicted that if the NES is active under steady state conditions, then inhibition of active nuclear export or disruption of the NES should cause EGFP-LC3 to become further enriched in the nucleus compared to the wild type protein. However, when we treated cells with LMB, an inhibitor of nuclear export, we found that this had essentially no effect on the subcellular distribution of EGFP-LC3 in either COS-7 or HeLa cells. Moreover, when we mutated the putative NES of LC3, EGFP-LC3 mNES was still found in both the cytoplasm and nucleus, a result inconsistent with a functional role of the NES. However, we also noted a second phenotype, in which the NES mutant was contained within punctate structures in the cytoplasm, presumably corresponding to autophagosomes or aggregates of LC3. Importantly, this phenotype was never observed in cells expressing wild type EGFP-LC3. This suggests that the region of the protein containing the putative NES, which is part of the ubiquitin fold of LC3, is important in regulation of the interactions of LC3 with other proteins or autophagosomes themselves.

Interestingly, beclin-1 contains an NES that, unlike that of LC3, is sensitive to inhibition of CRM-1 dependent transport by LMB treatment or mutation. Importantly, beclin-1's NES is also required for the protein's function in autophagy as well as its tumor suppressor activity [23]. Although our current studies did not reveal a specific function of LC3's putative NES in nuclear export, it is possible that the NES is active under other conditions not investigated here. The role of this presumed NES could be specific to the function of LC3, as analysis of the sequences of Atg8, GABA(A) receptor-associated protein (GABARAP) or Golgi-associated ATPase enhancer of 16 kDa (GATE-16) do not yield a predicted NES in this region for any of these LC3-related proteins. Despite the presence of a putative NES, LC3 does not contain a known NLS. LC3 could potentially harbor an as-yet unidentified NLS, or alternatively, its nuclear import could be mediated through an interaction with a protein containing an NLS.

Nucleocytoplasmic transport can occur by passive as well as active processes. We tested for passive transport by examining the rate of nucleocytoplasmic shuttling of EGFP-LC3 using a photobleaching assay. The results of these experiments revealed that compared to the rapid exchange of EGFP across the nuclear envelope, the rates of nuclear import and export of EGFP-LC3 are relatively slow. This suggests that an inhibitory mechanism may actually trap the protein in the nuclear and cytoplasmic compartments and prevent passive diffusion across the nuclear envelope. This could readily be accomplished if EGFP-LC3 formed a small oligomer or protein complex with a molecular weight of >60 kDa. Our observation that the diffusional mobility of EGFP-LC3 is nearly threefold slower than that of EGFP, as discussed further below, is consistent with this model. The ability of mutant Atg4B to sequester EGFP-LC3 in the cytoplasm [25], a finding confirmed here, may also reflect such a mechanism.

Our data do not fully rule out the possibility that the attachment of EGFP to LC3 alters the normal regulation of its nucleo-cytoplasmic transport, as attachment of a GFP tag has been noted to alter the nucleo-cytoplasmic distribution of other proteins [52]. However, EGFP itself lacks any nuclear import or export signals, and under the conditions of our experiments this protein alone is evenly distributed between the nucleus and cytoplasm. It is thus highly unlikely that the attachment of EGFP to LC3 could artificially cause LC3 to accumulate in the nucleus. Indeed, we could also detect endogenous LC3 in the nucleoplasm by immunofluorescence microscopy. Although at least one previous study has detected LC3-II in the nucleus by biochemical fractionation approaches [47], the nature of endogenous nuclear LC3 remains to be more fully investigated. Our current studies highlight the likelihood that this is not an artifact, but rather reflects the presence of a functional nuclear pool of LC3 in cells.

### Biological significance of the slow diffusional mobility of EGFP-LC3

The finding that the diffusional mobility of EGFP-LC3 in both the nucleus and cytoplasm is significantly slower than expected for a protein of its molecular weight can be explained by one of two possible models. The first model is that the protein undergoes transient binding events to cellular structures. This type of behavior has been best described for the case of nuclear proteins, which often showed slowed diffusion as the result of transient binding to DNA [42], [44]. Although to our knowledge LC3 has not been previously shown to interact with DNA, the slow diffusion of the protein in the nucleus could potentially indicate that this occurs. Similarly, the slower-than-expected diffusion of EGFP-LC3 in the cytosol could potentially be explained if EGFP-LC3 undergoes rapidly reversible binding to microtubules. However, a prediction of this “reversible binding” model is that a fraction of EGFP-LC3 should be visualized in its bound state on these structures, yet cytosolic EGFP-LC3 and tfLC3 appeared predominantly soluble (other than that bound to autophagosomes). Furthermore, it seems unlikely that binding of LC3 to different classes of structures in the nucleus and cytoplasm would coincidentally yield such similar *D*'s in the two compartments.

We thus favor a model in which EGFP-LC3 diffusion is slowed by its incorporation into macromolecular complexes in both the cytoplasm and nucleoplasm and that incorporation of the protein into these complexes prevents its rapid equilibrium across the nuclear envelope. Based on the Stokes-Einstein equation, the size of such complexes would be approximately 950 kDa in order to account for the 3-fold drop in *D* of EGFP-LC3 compared to EGFP in COS-7 cells. Although we do not yet know the identity of the components of these complexes, a number of candidates exist. For example the formation of LC3-II was shown to give rise to multimers [10]. Furthermore, LC3 also has several known binding partners, including human homologs of Atg7 and Atg3 [53], [54]. In addition, the Atg16L complex, an ∼800 kDa complex consisting of Atg16L and Atg12 conjugated to Atg5, determines the site at which LC3 lipidation occurs [55]. Interactions of LC3 with other proteins such as SOS1 have also been reported [56]. LC3 has also been reported to associate with the 3′ untranslated region of fibronectin mRNA and the 60S ribosomal subunit [57]. Very recently, LC3 was also shown to interact with a protein related to tumor protein 53-inducing nuclear protein 1 named TP53INP2 [24]. Normally a nuclear protein, TP53INP2 translocates to autophagosomes after rapamycin treatment or nutrient deprivation and is required for autophagy. Interestingly, studies using bioluminescence resonance energy transfer indicate that LC3 and TP53INP2 interact not only following induction of autophagy by starvation, but also to a lesser extent under steady state conditions when TP53INP2 is found primarily in the nucleus. Discerning which if any of these interactions accounts for the relatively slow diffusion of EGFP-LC3, and whether such complexes also account for the slow transport of EGFP-LC3 between the nucleus and cytoplasm will be an important goal for future studies.

### What is LC3 doing in the nucleus?

There is growing evidence that nucleocytoplasmic shuttling of proteins in the autophagy pathway contributes to the regulation of this process [23], [24]. We tested here whether upregulation of autophagy by amino acid starvation or rapamycin could be a physiological trigger of the transport of EGFP-LC3 out of the nucleus. In some published images of EGFP-LC3 in starved cells, the protein appears to remain enriched in the nucleus [20], [22], while others demonstrate a marked decrease in nuclear levels following starvation [18], [19]. We found that in both COS-7 and HeLa cells, soluble EGFP-LC3 remained enriched in the nucleus relative to the cytoplasm following upregulation of autophagy. Thus, induction of autophagy does not appear to specifically signal EGFP-LC3 to either exit or enter the nucleus *en masse*. However, in HeLa cells, the presence of soluble protein in either the nucleus or cytoplasm became progressively less obvious as the levels of EGFP-LC3 associated with autophagosomes following starvation increased. We speculate that the slow movement of EGFP-LC3 between the nucleus and cytoplasm reported by our photobleaching measurements is sufficient to allow for mass action to drive a steady loss of the protein from the nucleoplasm as the cytoplasmic pool of protein is incorporated into autophagosomes. Interestingly, we also observed a small, transient increase in nucleocytoplasmic ratio following amino acid starvation in HeLa cells, as well as a small but significant decrease in *D* for nuclear EGFP-LC3 after longer times of starvation. The physiological basis for these changes is not yet clear, but could reflect time-dependent changes in the interactions of LC3 with nuclear components, such as the putative complexes discussed above.

Although recent studies have focused on the role of LC3 in autophagy, other cellular functions of the protein have also been postulated. Originally identified as a microtubule associated protein [31], LC3 has also been shown to play a role in regulating the levels of fibronectin mRNA [57], [58]. In addition, LC3 interacts with SOS1, a guanine nucleotide exchange factor, leading to negative regulation of SOS1-dependent Rac1 activation of membrane ruffling [56]. The dendrite-specific Ca2+ sensing protein caldendrin has been identified as an LC3 interacting protein [59]. It will thus be of interest to determine in future studies which, if any of these functions involve a nuclear form of LC3.

## Materials and Methods

### Constructs

The plasmids encoding EGFP-LC3, tfLC3, EGFP-LC3^G120A^, and mStrawberry-Atg4B^C74A^ were the kind gift of Dr. Tamotsu Yoshimori, Osaka University [3], [14], [25]. The plasmid for EGFP was from Clontech. p53-EGFP was as previously described [42]. The Rev(68–90)GFP_2_-cNLS construct was the kind gift of Dr. Ralph Kehlenbach, Universität Göttingen [29]. Rev(68-90)GFP_2_-cNLS consists of the classic importin α/β-dependent NLS from SV40, two copies of EGFP, and the CRM1-dependent NES (residues 68–90) of HIV-1-Rev protein.

An NES mutant of LC3 (L63A L71A L73A), designated here as EGFP-LC3 mNES, was generated using a Quik Change site-directed mutagenesis kit (Stratagene, Inc.). Primers used to mutate amino acid 63 from a Leucine to an Alanine were 5′ C GTG AAT ATG AGC GAA **GCC** ATC AAG ATA ATT AGA AGG CGC 3′(forward) and 5′GCG CCT TCT AAT TAT CTT GAT **GGC** TTC GCT CAT ATT CAC G 3′ (reverse). This mutant was subject to further mutagenesis of amino acids 71 and 73 from Leucine to Alanine using the following primers; 5′ G ATA ATT AGA AGG CGC **GCG** CAG **GCC** AAT GCT AAC CAA GCC TTC 3′ (forward) and 5′ GAA GGC TTG GTT AGC ATT **GGC** CTG **CGC** GCG CCT TCT AAT TAT C 3′. All constructs were verified by sequencing using the CMV forward sequencing primer 5′CGC AAA TGG GCG GTA GGC GTG T 3′ by the Vanderbilt Sequencing Core.

### Cells and transfections

COS-7 cells were obtained from ATCC and maintained in DMEM supplemented with 10% fetal calf serum at 37°C and 5% CO_2_. For all experiments, cells were plated on coverslips two days prior to the experiment. Where indicated, the day prior to an experiment, cells were transfected using FuGene6 (Roche Diagnostics, Indianapolis) as recommended by the manufacturer. A HeLa cell line stably expressing EGFP-LC3 [18] was generously provided by Dr. Aviva M Tolkovsky, Cambridge Centre for Brain Repair. HeLa cells were maintained in RPMI supplemented with 10% fetal calf serum at 37°C and 5% CO_2_ and were plated on coverslips one to two days prior to experiments.

### Analysis of NES and NLS signals

Predicted NLS signals were screened using (http://cubic.bioc.columbia.edu/db/NLSdb/). An algorithm that predicts leucine-rich nuclear export signals was used to screen for predicted NES [26].

### Drug treatments and induction of autophagy

To inhibit active nuclear transport, cells were incubated in culture media containing 20 ng/ml leptomycin B (Sigma-Aldrich). To disrupt microtubules, cells were preincubated for 5 minutes on ice in phenol red-free DMEM containing 10% fetal calf serum and 50 mM HEPES. 5 µg/ml of nocodazole (NZ) (Sigma-Aldrich) was added and the cells were incubated for an additional 15 minutes on ice. They were then shifted to 37°C for 1 h in the continued presence of NZ, and either fixed or imaged live in the continued presence of NZ at 37°C [32]. Control experiments were performed using vehicle alone (DMSO). For starvation experiments, cells were washed 3 times with Earle's Balanced salt solution (EBSS) (Sigma-Aldrich). They were then incubated for the indicated times in EBSS at 37°C prior to imaging in the continued presence of EBSS. Rapamycin (Ready Made Solution, Sigma-Aldrich) was added to phenol red-free DMEM containing 10% fetal calf serum and 50 mM HEPES to a final concentration of 0.2 µM and cells were incubated at 37°C for 2, 4, or 6 h. As a control, cells were incubated with media containing an equivalent volume of vehicle (DMSO) for 6 h. Fixed cells were mounted using Fluoromount G supplemented with 25 mg/ml DABCO (1,4 diazabicyclo[2.2.2]octane) (Sigma-Aldrich) and allowed to solidify overnight prior to imaging.

### Immunofluorescence staining

A rabbit antibody against recombinant human LC3B (residues 1–120) was from Medical & Biological Laboratories Co, Ltd, (Nagoya, Japan). Mouse anti-GFP was from Invitrogen Molecular Probes (Carlsbad, CA). Fluorescently labeled secondary antibodies were purchased from Jackson ImmunoResearch Laboratories Inc. (West Grove, PA).

Cells were fixed in 3.7% PFA for 15 min at RT followed by permeabilization with either PBS containing 10% FCS and 0.1% saponin for 30 min or 0.1% ice-cold TX-100 in PBS for 5 min on ice. After blocking they were then incubated with primary antibodies (1∶200 dilution) for 30 min at RT. Cells were then washed, incubated with 1 µg/ml Cy3 anti-mouse or anti-rabbit secondary antibodies (Jackson ImmunoResearch Laboratories) for 30 min at RT, and washed again. Coverslips were then mounted on slides using Fluoromount G supplemented with 25 mg/ml DABCO (1,4 diazabicyclo[2.2.2]octane) (Sigma-Aldrich) and allowed to solidify overnight prior to imaging.

### Confocal microscopy and photobleaching experiments

Fluorescence images were obtained using a Zeiss LSM 510 confocal (Carl Zeiss, Thornwood, NY). Images were collected using a 40X 1.3 NA Zeiss Plan-Neofluar objective or 100X 1.4 NA Zeiss Plan-Apochromat objective. The confocal pinhole was set at 1 Airy unit. Cells were maintained in phenol-red free DME containing 10% fetal calf serum and 50 mM Hepes or EBSS where indicated for live-cell imaging experiments. Cells were imaged live at 22°C for quantification of N/C ratios or 37°C for FRAP analysis using a stage heater and objective heater. EGFP fluorescence was excited using the 488 nm line of a 40 mW Argon laser, and mRFP and mStrawberry were excited at 543 nm of a HeNe laser and detected using filter sets provided by the manufacturer. For presentation purposes, images were exported as TIFF files and contrast was adjusted using Adobe Photoshop.

For FRAP measurements of nuclear import and export, cells were imaged using a 40X 1.3 NA Zeiss Plan-Neofluar objective at 3X digital zoom with the confocal pinhole set to 1 Airy unit. Images were collected at 1% transmission with line averaging of 4. For measurements of nuclear import, an oval completely filling the nucleus was selected as the bleach region. For measurements of nuclear export, the bleach region contained the cytoplasmic region but excluded the nucleus. Photobleaching was accomplished by transiently increasing the laser power to 100% and repetitively scanning the bleach region either 10 (EGFP-LC3 and tfLC3) or 40 (EGFP) times. Images were collected every 5 s during the recovery phase for measurements of nuclear import and every 5 s (for EGFP) or 8 s (for EGFP-LC3, tfLC3) for measurements of nuclear export.

FRAP measurements were performed at 37°C. For FRAP measurements of lateral diffusion, cells were imaged using a 40X 1.3 NA Zeiss Plan-Neofluar objective at 4X digital zoom with the confocal pinhole set to 1 Airy unit. For cells expressing EGFP, the location of the nucleus was identified by focusing to the top of the cell. Only the EGFP channel was imaged for cells expressing tfLC3. Images were collected for a square region 70×70 pixels (7.84 µm×7.84 µm). Photobleaching of a circular bleach region 25 pixels in diameter (2.75 µm) was performed by repetitively scanning the bleach region 20 times using 100% laser power. Images were collected every 0.14 s during the recovery phase for a total of 16.75 s. Under the conditions of these experiments, no overall photobleaching due to imaging was observed. For presentation purposes, recovery curves from 5–7 cells from an individual experiment were averaged.

### Quantification of N/C ratios from confocal images

LSM images were exported as Tiff files and fluorescence intensities in the nuclear and cytoplasmic regions were quantified using Image J (http://rsbweb.nih.gov/ij/). Background-corrected N/C ratios were calculated from mean fluorescence intensities measured within a small square or circular region of interest placed within the nucleus, cytoplasm, and outside of each cell. Measurements of EGFP-LC3 in the cytoplasm were made in regions of the cell devoid of punctate structures. Note that the absolute N/C values varied somewhat between experiments depending on exact imaging conditions and thus comparisons were only made for paired experiments. Where indicated, N/C data were normalized to control values for a given experiment to enable comparison across different days.

### FRAP analysis

To analyze the FRAP data, we used a modified form of the Gaussian laser FRAP equation of Axelrod [60] that corrects for diffusion of molecules during the photobleach [46]. The theoretical basis for this approach is described elsewhere [61]. In brief, in this approach, we used an experimentally determined radius, *r_e_*, obtained immediately after the photobleach, to calculate diffusion coefficients. To measure *r_e_*, the LSM 510 software was used to calculate a pixel-wide line profile across the box containing the bleaching spot. We averaged the intensities from 4 profile measurements from each dataset. The intensity profiles were then fit to a postbleach profile by the Gaussian laser to obtain the half width at *e* ^−2^ height of postbleach intensity distribution from
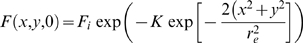
(eq.1)


Assuming *r_n_* ≤ *r_e_*, the following the calculation was performed as described in [60]

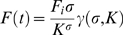
(eq.2)


where 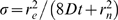 and 
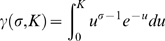
, the lower incomplete gamma function. The bleaching parameter (*K*) was computed from initial fluorescence intensity, *F*(0), by solving



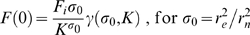
(eq.3)


Note that when *r_n_*  =  *r_e_*, then Eq. (1)∼(3) reduce to the conventional FRAP formula as described in [60].

In some instances FRAP curves were analyzed individually then the resulting *D* and Mf averaged, whereas for others data analysis was performed on averaged FRAP curves collected from 10 different cells on a single day rather than individual recovery curves to improve the signal to noise ratio [38]. Mobile fractions were calculated as described previously [32].
